# Vertical Distribution of Lead and Mercury in the Wetland Argialbolls of the Sanjiang Plain in Northeastern China

**DOI:** 10.1371/journal.pone.0124294

**Published:** 2015-04-20

**Authors:** Chunye Lin, Peizhong Li, Hongguang Cheng, Wei Ouyang

**Affiliations:** State Key Joint Laboratory of Environmental Simulation and Pollution Control, School of Environment, Beijing Normal University, Beijing, 100875, China; Nanjing University, CHINA

## Abstract

The wetland Argialbolls pedon was chosen to investigate the effects of pedogenic processes and anthropogenic activities on the vertical distribution of lead and mercury concentration and to assess the potential use of soil as an archive of atmospheric Pb and Hg pollution. The soil was sampled from 5 cm from the surface to a depth of 90 cm at two locations in the Sanjiang Plain in northeastern China. The soil was analyzed for pH, soil organic matter (SOM), Fe, Mn, and Al. The results indicate that the SOM concentration gradually decreased with depth, while Fe and Mn were reductively leached from the upper horizons and accumulated significantly in the lower argillic horizons. Atmospheric Pb and Hg deposition and their redistribution during the pedogenic process led to a unique vertical distribution in the wetland Argialbolls. Overall, Pb was leached from the upper horizons and then accumulated in the lower argillic horizons. However, the Hg concentration decreased with depth, following the SOM distribution. The Pb concentration was significantly correlated to the Fe and Mn concentrations in the Argialbolls profiles, while the Hg concentration was significantly correlated with SOM. Post-depositional mobility along the wetland Argialbolls profile is higher for Pb and low for Hg. Therefore, the Argialbolls profile does not provide an accurate reconstruction of atmospheric Pb deposition, but might provide an accurate reconstruction of net atmospheric Hg deposition.

## Introduction

Wetlands are areas that are sporadically or permanently flooded; and develop anoxic conditions and subsequent soil bioreduction [1]. In wetland soils, trace metal/metalloid mobility depends on variables such as redox potential (Eh) and pH, organic matter, and Fe/Mn oxides content [2]. Oxidative sediment conditions generally stabilize trace metals in Fe/Mn oxides, while reduction and the concomitant desorption of organic matter release trace metals/metalloids into the soil solution with subsequent downward leaching [2–4]. Therefore, vertical distributions of trace metals in soils depend on both pedogenic processes and the influences of anthropogenic activity.

While many studies have analyzed the vertical distributions of Pb and Hg in soils, most focused on forest soils and the soils near nonferrous mining and smelting. The concentrations of Pb and Hg usually decreased with depth in the studied soils near smelting operations due to their high atmospheric deposition [5–7]. In forest soils, however, the vertical distribution of Pb and Hg concentrations are more complicated, depending on soil properties and atmospheric metal deposition [8–13].

On the other hand, some studies determined the vertical distribution of elemental concentrations in peat and sediment profiles in order to retrieve the past atmospheric deposition of trace metals [14,15]. However, fewer studies have been performed regarding the vertical distributions of elements in wetland Argialbolls. An example is the Sanjiang Plain of northeastern Heilongjiang Province of China, where the wetlands are a major natural ecosystem and are generally Argialbolls.

The objective of this study is to investigate the Pb and Hg concentration profile for wetland Argialbolls in the Sanjiang Plain of northeastern China. This information is important to understanding the mobility and redistribution of anthropogenic Pb and Hg via atmospheric deposition during pedogenic processes in Argialbolls and to assess the potential use of the soil as an archive of atmospheric Pb and Hg pollution.

## Materials and Methods

### Ethics Statement

Wetland soil cores were collected with single gouge augers (Eijkelkamp) from the natural wetland in late May 2010. This field area does not require any permits or approvals of any authorities and does not involve endangered or protected species. This wetland is not privately owned or protected. The field site extends from134°5′0″ to 134°7′30″0 and 47°4′20″ to 47°4′30″.

### Description of Study Site

The study site was described in our previous paper [16] and thus briefly introduced here. It is located in the Sanjiang Plain of the Heilongjiang Province, China. The annual average temperature and precipitation are 3°C and 500 to 600 mm, respectively. Water and soil are completely frozen from late October to early April. A major water system consists of the Amur, Songhua, and Ussuri Rivers which provide alluvial deposits in this area. Wetland soil in the Sanjiang Plain is generally classified as Histosols (Argialbolls) [17], with organic matter accumulation in the A horizon, eluviation of clay minerals from the A and E horizons (albic horizon) and their illuviation in the B horizon (argillic horizon). The major pedogenic process is wetting-drying cycles that lead to alternation of oxidation-reduction processes [18].

### Soil Core Collection and Analysis

In May 2000, two 95 cm deep soil cores were collected with single gouge augers (Eijkelkamp) from the natural wetland (located at approximately 134.0884E and 47.4036N), which is generally not impacted by local anthropogenic activity. The distance between the two soil cores is approximately 500 m. The cores were cut into 5 cm slices. The soil samples were transferred to acid-washed dark-colored polyethylene bags and were freeze-dried, slightly crushed, passed through a 2 mm sieve and stored in glass bottles [16].

The soil analysis of pH, organic matter, Al, Fe, and Mn was described in our previous paper [16] and thus briefly introduced here. The soil organic matter content was determined by weight loss on ignition to 400°C [19]. The Pb was measured with ICP MS (X Series II, Thermo Electron) following the soil digestion with HNO_3_–HF–HClO_4_ [20]. Portions of the soil samples were digested with aqua regia, 1% KMnO_4_ solution, and 1% oxalic acid. The Hg in the supernatant was determined by cold vapor atomic fluorescence spectrometry (CVAFS).

In addition, four reference soils (GSS17, 19, 25, and 26) (triplicate), provided by the Institute of Geophysical and Geochemical Exploration, Chinese Academy of Geological Sciences, were digested and analyzed to assess the analytical quality. Average relative errors were -7.9~-1.2%, -3.4~-1.2%, -1.2~2.3%, -2.6~4.8%, and -2.6~2.4% for Al, Fe, Mn, Pb, and Hg, respectively.

## Results and Discussions

### General Properties of the Argialbolls Profiles

**Fig 1:**
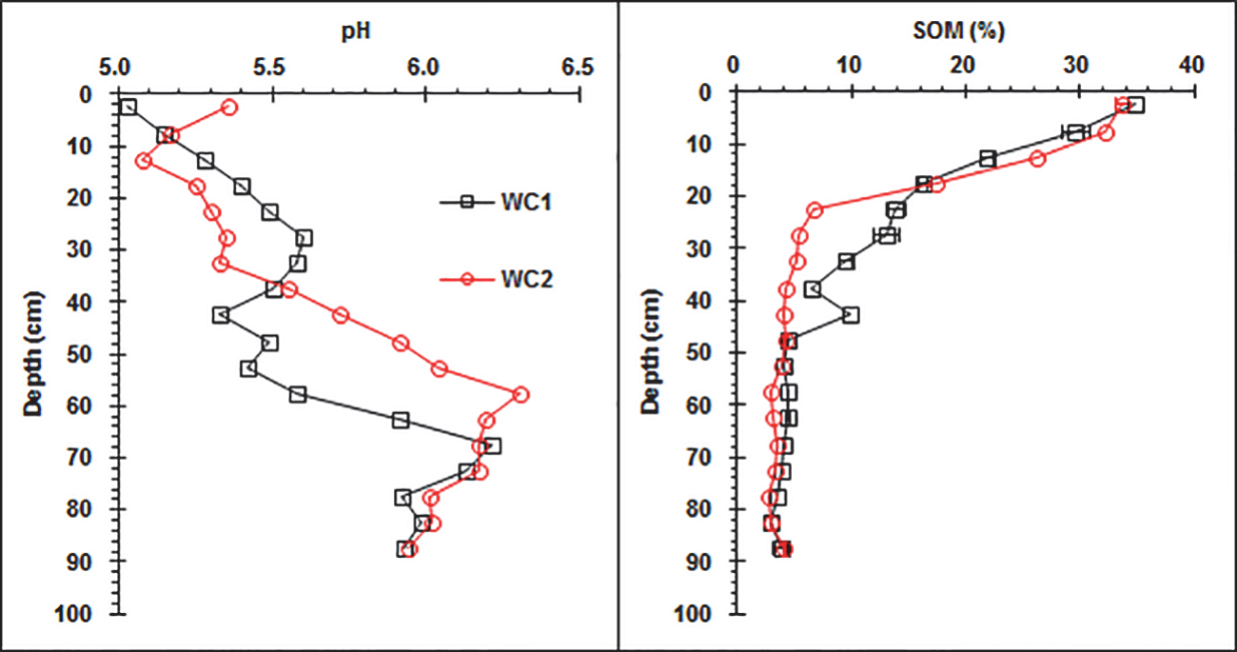
Vertical distributions of pH and soil organic matter (SOM) along the wetland Argialbolls profile (WC1: wetland core one, WC2: wetland core two).

The pH for wetland soil profiles generally increased from 5.0~5.5 in the surface horizon to 6.0~6.5 in the bottom horizon, indicating that the soil was slightly acidic (Fig 1). Acidic conditions usually favor the leaching of heavy metals from soils [21].

The SOM concentration in the wetland soil generally decreased from approximately 34% at a 0 to 5 cm depth to approximately 4% at a 45 to 50 cm depth (Fig 1). Below 50 cm, the SOM concentration in the wetland soil generally did not change with depth. The vertical distribution of the SOM content is similar to that of soil organic carbon in the Sanjiang Plain, reported by Chi et al. [22].

**Fig 2:**
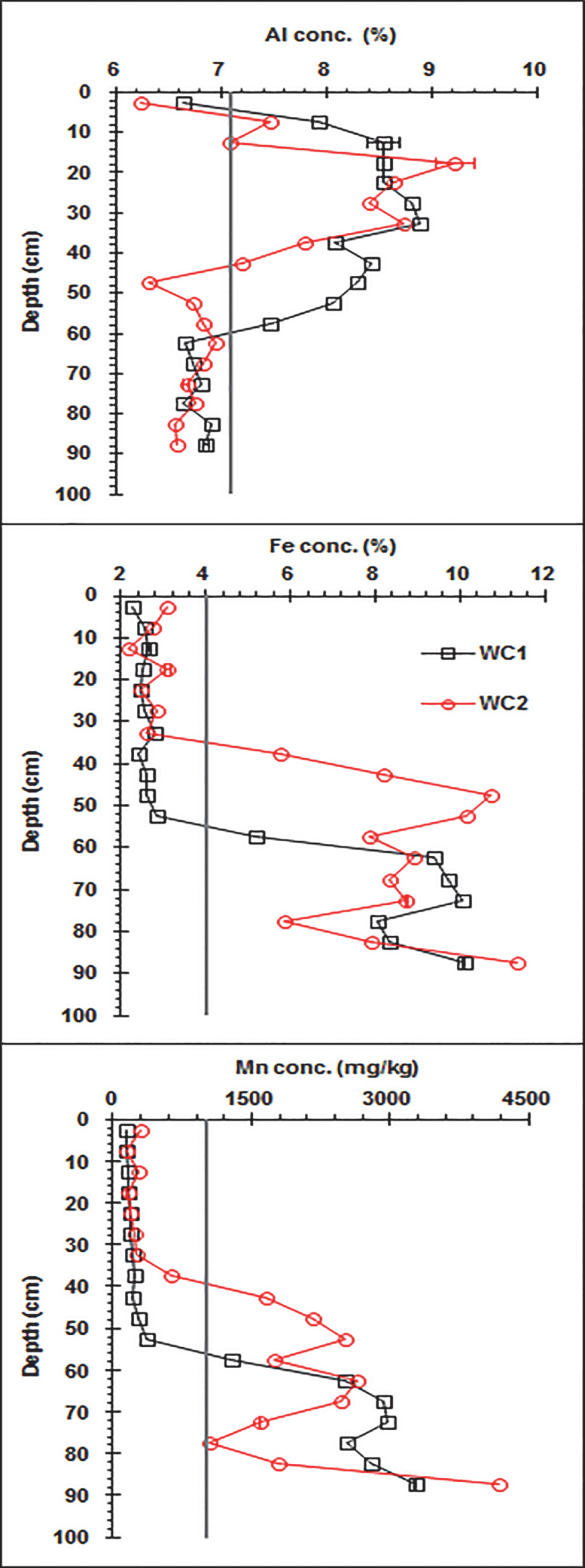
Vertical distributions of the Fe, Mn, and Al concentrations along the wetland Argialbolls profile (WC1: wetland core one, WC2: wetland core two). The vertical line represents the median concentration for worldwide soils.

In contrast to SOM, Fe and Mn accumulated in the lower horizons of the wetland soils (Fig 2). In the upper horizons of the wetland soil (0 to 55 cm depth for core 1 and 0 to 35 cm depth for core 2, where SOM generally accumulated), which were low in Fe and Mn oxides, Fe and Mn concentrations were only approximately 2.2 to 2.8% and 145 to 281 mg kg^-1^, respectively, lower than the median concentrations of Fe and Mn for worldwide soils [23]. However, in the Fe and Mn oxide-rich lower horizons of the wetland soil (55 to 90 cm depth for core 1 and 35 to 90 cm depth for core 2), Fe and Mn concentrations were generally 5.2 to 11.3% and 1048 to 3473 mg kg^-1^, respectively, higher than the median concentrations of Fe and Mn for worldwide soils [23].

The profile of the Al concentration was different from both the Fe and Mn and the SOM concentration profiles. The Al concentration initially increased and then decreased with soil depth, with the higher concentration in the middle-upper horizons, indicating that Al was not leached.

The vertical distributions of Al, Fe, Mn, and SOM concentrations in the wetland Argialbolls demonstrated that Fe and Mn were reductively leached from the upper horizons and accumulated in the lower horizons. Similar vertical distributions of Fe and Mn concentrations were observed in the Dystric Cambisol profile of Austria, where Fe and Mn contents increased from 2.8% and 160 mg kg^-1^ at 1.5 cm depth to 11.1% and 1596 mg kg^-1^ at 200 cm depth, respectively, due to long-term weathering and leaching [24].

### Pb and Hg Concentration Profiles for the Wetland Argialbolls

**Fig 3:**
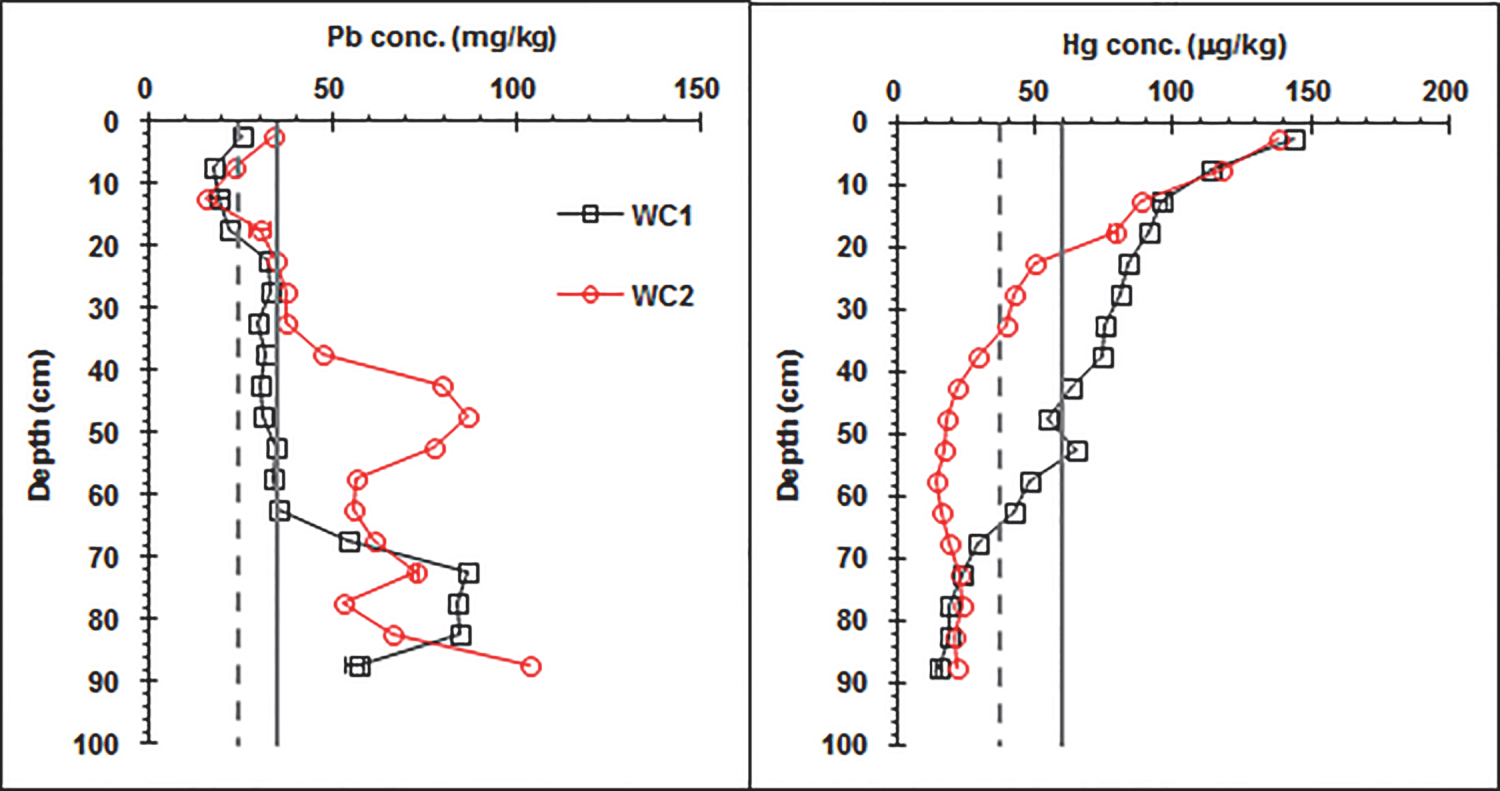
Vertical distributions of the Pb and Hg concentrations along the wetland Argialbolls profile (WC1: wetland core one, WC2: wetland core two). The vertical line and dot line represent the median concentration for worldwide soils and the average concentration in the soil A horizon for the Heilongjiang Province, respectively.

In general, the Pb concentration profile was similar to the Fe and Mn concentration profiles, while the Hg concentration profile was similar to the SOM concentration profile (Fig 3).

Generally, the concentration of Pb in the upper SOM-accumulated horizons was lower than that in the low Fe/Mn oxide-accumulated horizons (Fig 3 and S1 Table). In addition, the concentration of Pb in the upper SOM-accumulated horizons was lower than the median concentration of Pb for worldwide soils and similar to the average concentration of Pb in the A horizon of the Heilongjiang Province, while the concentrations of Pb in the low Fe/Mn oxide-accumulated horizons was higher than them [23,25].

A similar Pb distribution to our study was observed in Norwegian Podzols, where atmospheric Pb was partially concentrated in the humus layer and partly enriched along with Fe in the B horizon due to leaching from the polluted organic surface soil [8]. The importance of spodic horizons to Pb accumulation in the mineral soil was also observed in the northeastern U.S. [26,27]. In a forest Luvisol profile, however, Pb concentration decreased with depth, suggesting the binding of Pb to soil organic matter [28]. Trace metal release in wetland soils was controlled by organic matter mobility and the reduction of Fe-oxyhydroxides [3]. Despite the relative immobility of metals in soils due to trapping by organic matter, the potential for mobility is still present [6,9,10]. Rapid transport of anthropogenic Pb (approximately 1 cm yr^-1^) was measured through soils in southeast Missouri [6], while the Pb migration rate is estimated at approximately 0.01 cm yr^-1^ in a complex temperate soil profile [10].

In general, the concentration of Hg in the upper SOM-accumulated horizons was higher than in the lower Fe/Mn oxide-accumulated horizons (Fig 3 and S1 Table). The concentration of Hg in the upper SOM-accumulated horizons was higher than the median concentration of Hg for worldwide soils and the average concentration of Hg in the A horizon of the Heilongjiang Province, while the concentration of Hg in the low Fe/Mn oxide-accumulated horizons was generally lower than them [23,25]. In addition, the concentration of Hg at a 20 to 65 cm soil depth was higher for WC1 than for WC2, possibly due to the differences in SOM content and composition and other soil properties between the two cores.

A similar Hg distribution was reported for the peat core from Xiaoxing’an Mountain, adjacent to the Sanjiang Plain, where the concentration of Hg gradually decreased from approximately 185 to 66 μg kg^-1^ at a depth of 0 to 55 cm [29]. Remote upland forest soil cores from southwestern China have also recorded the atmospheric depositional history of Hg [11]. For the forest Inceptisols in the northeastern U.S., the Hg concentration decreased with increasing depth, following the SOM distribution, but for the forest Spodosols the Hg concentration in the Bs horizons was significantly higher than that in all other mineral horizons [12]. Podzolization, the process of SOM complexing and eluviating Fe and other metals from the E horizon to Bs horizon, is likely to be responsible for the elevated Hg and other metal accumulation in the Bs horizon [27,30]. Gaseous elemental Hg has a long residence time in air, which favors long-range transport and homogenization on a hemisphere scale via the atmosphere [14]. In addition, cold climate promotes enhanced storage of Hg in the peat bog [14].

Large volumes of trace elements have been produced and dispersed into the pedosphere [31–35], and potential cumulative anthropogenic inputs in world civilization-relevant land surface soil up to the year 2000 were 0.21 mg kg^-1^ for Hg and 75.8 mg kg^-1^ for Pb [33]. The vertical distributions of Pb and Hg in soil profiles depend on their atmospheric deposition rates or loads, soil properties, and pedogenic processes. We found that SOM has significantly accumulated in the upper horizons of the wetland Argialbolls, while Fe and Mn have been significantly leached from the upper horizons and accumulated in the low horizons. These pedogenic processes have indeed resulted in the unique vertical distribution of Pb and Hg in the wetland Argialbolls. Overall, the post-depositional mobility is higher for Pb and lower for Hg in the wetland Argialbolls profile.

### Relationships between Fe, Mn, SOM, Pb, and Hg

**Fig 4:**
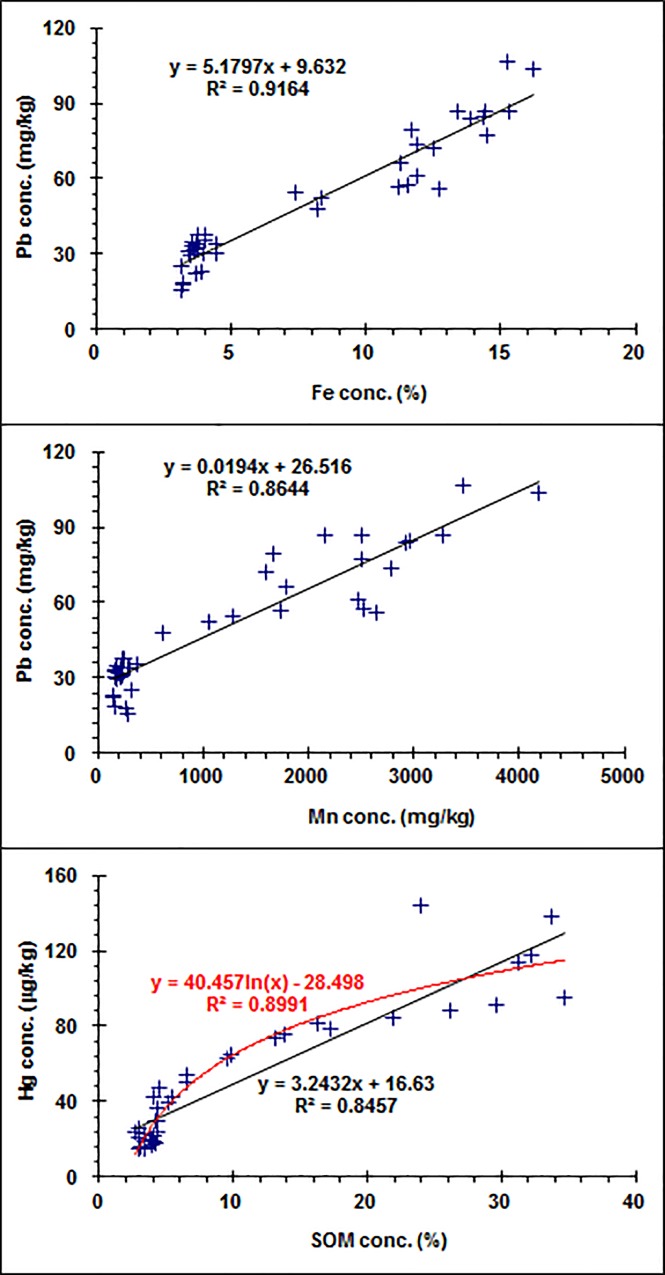
Correlations between Pb and Fe, Pb and Mn, and Hg and soil organic matter (SOM) for the Argialbolls profiles.

Correlation analysis further demonstrated that the concentration of Pb in the soil cores was significantly positively correlated with the concentrations of Mn and Fe (*p*<0.001), while Hg was significantly positively correlated with SOM (*P<0*.*001*) (Fig 4).

Previous studies showed that the Hg concentration in soil profiles was correlated with SOM. In the Tibetan forest soil profile, the Hg concentration was significantly correlated with SOM, showing that SOM is a key variable for storage and vertical distribution of Hg in the soil [13]. In the remote upland forest of southwestern China, the Hg concentration in soil profiles was highly correlated to SOM [11]. In tropical Oxisols and Ultisols profiles, however, Hg concentration was correlated to Fe or clay concentrations, probably due to the high affinity of Hg for these carrier phases [9]. A strong linear relation was evident at the McTier Creek watershed, but at the Fishing Brook watershed, the linear relationship was weak [36]. Therefore, the relationship between Hg and SOM in soils mainly depends on the soil types.

The vertical distribution and correlation analysis indicated that Fe/Mn oxides might be the primary control on the distribution of Pb in the wetland Argiabolls, while the distribution of Hg might be primarily controlled by SOM. The concentration of Pb shows that there may be two episodes: accumulation and leaching in the wetland soil of the Sanjiang Plain. The increase in their concentration in the lower Fe/Mn oxide-accumulated horizons may be due to their post-depositional mobility and redistribution via long-term leaching in the course of soil formation. However, the upward increase of Pb concentration at a 0 to 10 cm depth may be due to atmospheric deposition and the partial retention of anthropogenic Pb in this topsoil. Similar results were reported by Prohaska et al. [24,37]. They observed Fe, Mn, Cu, Pb, and Cd leaching from the upper part of soil profile and their subsequent accumulation in the lower part of the soil profile. The larger concentrations in the uppermost part of the soil profile compared to the middle are most likely due to anthropogenic Cu, Pb, and Cd input during the last few decades [24,37]. Hansson et al. also observed the downward leaching of atmospherically deposited trace metals in peat [38–40].

## Conclusions

In the wetland Argialbolls profiles, Fe and Mn oxides mainly control the vertical distribution of Pb, while SOM primarily controls the vertical distribution of Hg. The accumulation of SOM in the upper horizons and Fe and Mn oxides in the lower horizons led to the unique vertical distribution of Pb and Hg in the wetland Argialbolls. Overall, the post-depositional mobility is higher for Pb and lower for Hg along the wetland Argialbolls profile. Therefore, the Argialbolls profile does not provide an accurate reconstruction of the atmospheric Pb deposition, but might provide an accurate reconstruction of the net atmospheric Hg deposition.

## Supporting Information

S1 Table The concentrations of Hg and Pb in the wetland Argialbolls core 1 (WC1) and core 2 (WC2) of the Sanjiang Plain. (DOCX)
