# Supplementary material for: Vertical Distribution of Lead and Mercury in the Wetland Argialbolls of the Sanjiang Plain in Northeastern China
Source: PLoS One. 2015 Apr 20;10(4):e0124294. doi: 10.1371/journal.pone.0124294 (PMC4403988; doi:10.1371/journal.pone.0124294)
Supplement: S1 Table — (DOCX) [file pone.0124294.s001.docx]

S1 Table. The concentrations of Hg and Pb in the wetland Argialbolls core 1 (WC1) and core 2 (WC2) of the Sanjiang Plain.

| Depth (cm) | Hg (mg/kg) | |  | Pb (mg/kg) | |
| --- | --- | --- | --- | --- | --- |
|  | WC1 | WC2 |  | WC1 | WC2 |
| **0-5** | 144.0 | 138.0 |  | 25.2 | 33.7 |
| **5-10** | 114.0 | 117.5 |  | 17.7 | 23.0 |
| **15-15** | 95.5 | 88.0 |  | 18.8 | 15.3 |
| **15-20** | 91.0 | 78.5 |  | 22.0 | 30.3 |
| **20-25** | 84.0 | 50.0 |  | 32.4 | 34.5 |
| **25-30** | 81.0 | 42.0 |  | 33.4 | 37.5 |
| **30-35** | 75.5 | 39.5 |  | 29.4 | 37.3 |
| **35-40** | 74.0 | 29.5 |  | 31.5 | 47.5 |
| **40-45** | 63.0 | 22.0 |  | 30.2 | 79.6 |
| **45-50** | 54.5 | 18.0 |  | 30.8 | 87.0 |
| **50-55** | 65.0 | 16.5 |  | 34.2 | 77.3 |
| **55-60** | 47.5 | 14.5 |  | 34.1 | 56.5 |
| **60-65** | 42.5 | 16.0 |  | 35.0 | 55.7 |
| **65-70** | 29.5 | 19.0 |  | 54.1 | 61.3 |
| **70-75** | 23.5 | 22.5 |  | 86.6 | 72.4 |
| **75-80** | 18.5 | 23.5 |  | 83.7 | 52.5 |
| **80-85** | 18.5 | 20.5 |  | 84.5 | 66.2 |
| **85-90** | 15.0 | 22.0 |  | 57.3 | 103.6 |
